# The effect of polymorphism in the *FADS2* gene on the fatty acid composition of bovine milk

**DOI:** 10.5194/aab-62-547-2019

**Published:** 2019-09-18

**Authors:** Witold Stanisław Proskura, Michał Liput, Daniel Zaborski, Zbigniew Sobek, Yu-Hsiang Yu, Yeong-Hsiang Cheng, Andrzej Dybus

**Affiliations:** 1 Laboratory of Molecular Cytogenetics, Department of Ruminants Science, West Pomeranian University of Technology, Szczecin 71-270, Poland; 2 Laboratory of Biostatistics, Department of Ruminants Science, West Pomeranian University of Technology, Szczecin 71-270, Poland; 3 Department of Genetics and Animal Breeding, Poznań University of Life Sciences, Poznań 60-637, Poland; 4 Department of Biotechnology and Animal Science, National Ilan University, I-Lan 260, Taiwan

## Abstract

Polyunsaturated fatty acids (PUFAs) play a role in a wide variety of physiological processes. They are produced by a series of desaturation and elongation reactions. Δ-6-desaturase is a membrane-bound enzyme that catalyzes the conversion of α-linolenic acid (C18:3n-3) and linoleic acid (C18:2n-6) to stearidonic acid (18:4n-3) and γ-linolenic acid (18:3n-6). It is encoded by the *FADS2* gene located on bovine chromosome 29. The aim of this study was to identify a single nucleotide polymorphism in the *FADS2* gene and to determine possible associations with milk fatty acid composition in two breeds of dairy cattle, i.e., Jersey and Polish Holstein-Friesian. Direct DNA sequencing revealed the presence of an A-to-G substitution in intron 3 of the *FADS2* gene (rs209202414). Both populations were genotyped with an appropriate PCR-RFLP assay. The following genotype distributions were observed: for Jerseys, *AA* = 0.24, *AG* = 0.63, and *GG* = 0.13; for Polish Holstein-Friesians, *AA* = 0.17, *AG* = 0.40, and *GG* = 0.43. In Jerseys, statistically significant relationships were found between the *FASD2* genotypes and the following milk fatty acids: lauric (P=0.0486), behenic (P=0.0199), lignoceric (P=0.0209), oleic (P=0.0386), eicosatrienoic (P=0.0113), and docosadienoic (P=0.0181). In Polish Holstein-Friesian cows, significant associations were observed for erucic (P=0.0460) and docosahexaenoic (P=0.0469) acids. The study indicated the A-to-G substitution (rs209202414) in the bovine *FADS2* gene as a potential genetic marker for fatty acid composition in cattle milk.

## 1 Introduction

The feeding of dairy cows is the main factor impacting milk fat composition. Pasture intake reduces the concentration of saturated fatty acids (SFAs) in the milk of grazing cows (Couvreur et al., 2007; Frigo et al., 2015; Hanuš et al., 2016; Ponnampalamet al., 2018). Furthermore, genetic factors influence fatty acid (FA) variability. The FA profile in milk changes during lactation, emphasizing the relationship between the physiological status of cow and milk composition (Bastin et al., 2011). The effects on milk FA composition are also breed-dependent. The greatest breed differences are observed between Holstein and Jersey milk (with the higher concentrations of SFAs in Jerseys) (Arnould and Soyeurt, 2009). Some authors have reported that milk fat composition is modulated by the polymorphisms in genes involved in milk fat synthesis processes, like *DGAT1* and *SCD1* (Carvajal et al., 2016, Tzompa-Sosa et al., 2016).

Dietary long-chain polyunsaturated fatty acids (PUFAs) increased intestinal *FADS2* mRNA abundance but had modest effects on its level in the liver of suckling pigs (Jacobi et al., 2011). PUFAs regulate fatty acid desaturase (FADS1, FADS2) activity in the liver and adipocyte tissue (Nakamura and Nara, 2004; Ralston et al., 2015). Hatanaka et al. (2016) reported that long-chain polyunsaturated fatty acid (LC-PUFA, > C20) intake is crucial for the growth of δ-6-desaturase knockout (D6D-KO) mice. The *FADS2* indel polymorphism in the European grayling was found to be associated with muscle FA composition (Renaville et al., 2013). Matsumoto et al. (2014) found that the SNP (g. -823G > A) in the *FADS2* promoter had a significant effect on several beef quality traits, including beef marbling score, whereas Takahashi et al. (2016) reported a highly significant association between the rs211580559 SNP in exon 7 of the *FADS2* gene and intramuscular C18:2(n-6) composition. In the transcriptomic study, Wang et al. (2017) pointed to *FADS2* as a strong candidate gene that may be associated with intramuscular fat deposition. Recently, Gol et al. (2018) reported that the polymorphism in the porcine *FADS2* gene is linked to arachidonic acid metabolism.

Fatty acid desaturase-2 (FADS2) is a component of the lipid metabolic pathway and converts essential FA into LC-PUFA by the introduction of a double bond between carbon atoms at positions Δ6 and Δ7 of FA (14). FADS2 is a rate-limiting enzyme involved in the conversion of linoleic acid (LA; 18:2n-6) into γ-linolenic acid (GLA; 18:3:n-6) and that of α-linolenic acid (ALA; 18:3n-3) into stearidonic acid (SDA; 18:4n-3).

Some genome-wide association studies showed that the *FADS* locus is one of the strongest genetic predictors of plasma phospholipid PUFA (Lemaitre et al., 2011; Tanaka et al., 2009). Ibeagha-Awemu et al. (2014) demonstrated positive associations between three SNP within the *FADS2* gene and the milk PUFA in Canadian Holstein cows. Therefore, the main aim of this study was to analyze the associations between the *FADS2* gene polymorphism and milk fat composition in two breeds of dairy cattle (Polish Holstein-Friesian and Jersey).

## 2 Materials and methods

### 2.1 Animals

The study involved 150 Holstein-Friesian cows housed in a conventional free-stall barn in West Pomeranian Province, Poland, and 104 Jersey cows kept in a tie-stall barn in Greater Poland Province. Only healthy animals from 2–5 years old were included. The nutrition and management of cows were quite similar. Feeding was based on a total mixed ration (TMR), mainly composed of maize silage, grass haylage, maize cereals, oat cereals, soybean meals, and mineral–vitamin mixtures. No ethical consent was required for the present study since the milk samples were collected during milking and the blood samples during routine veterinary visits.

### 2.2 SNP identification and genotyping

Genomic DNA was isolated from whole peripheral blood using the salting-out method (MasterPure^™^ DNA Purification Kit for Blood, Epicentre, Madison, Wisconsin, USA). Exons 1, 3, and 12 of the bovine *FADS2* gene were amplified using the primers given in Table 1. The reference sequence of the *FADS2* gene located on chromosome 29 (GenBank Acc. No. NC_037356.1) was used.

**Table 1:** Primer sequences used for the amplifications of the bovine *FADS2* exons (1, 3, and 12).

Region	Primer sequences (5${}^{\prime }$–3${}^{\prime }$)	Product	Annealing
		length	temp. (${}^{\circ }$C)
Exon 1	F1: GGAGGAGAAGACAAAAGCCGA	437	60
	R1:TGAGCGCCGTAGACACTTTT		
Exon 3	F3: TCCCAGATCACCGAGGACTT	292	60
	R3: TTCAGAGCGTTGGCACCTAG		
Exon 12	F12: CGGGCAACTGGTCCCTTTAT	389	60
	R12: GTCCCATGACCAAGTGCCTC		

PCR amplifications were performed in a total volume of 15 µL containing 50 ng of genomic DNA, 1.5 mM ${\mathrm{MgCl}}_{2}$, 0.2 mM of each dNTP, 15 pmol of each primer, and 0.3 U of Taq polymerase (Eur${}_{x}$, Poland). The following thermal profile was applied: 5 min at 94 ${}^{\circ }$C, 32 cycles of 30 s at 94 ${}^{\circ }$C, 30 s at the annealing temperature, and 30 s at 72 ${}^{\circ }$C; and a final extension of 5 min at 72 ${}^{\circ }$C. The PCR products were separated in agarose gel (1 %, 30 min, 120 V) and then extracted using the GEL/PCR Purification GPB Mini Kit (GenoPlast Biochemicals, Poland). Finally, the samples were sent for sequencing to an external laboratory (Genomed, Poland). A PCR-RFLP assay has been developed for the genotyping of an A-to-G substitution (rs209202414) in intron 3 of the *FADS2* gene. The PCR conditions were the same as those described above (Primers F3 and R3, Table 1). A total of 10 µL of the PCR product was digested with 2 U of *Tse*FI restriction enzyme (SibEnzyme Ltd, Russia). Subsequently, the restriction fragments were separated in a 3 % agarose gel (60 min, 120 V) stained with ethidium bromide.

### 2.3 Milk samples and fatty acid composition

Milk samples for the determination of fatty acid composition were collected from cows after the 90th day of lactation to avoid the period of negative energy balance and to maximize the period of de novo milk fat synthesis in the mammary gland. The samples were transported to the laboratory and kept frozen until further processing. Total lipids were extracted from each sample using a chloroform–methanol solution according to Folch et al. (1957). FAs were transformed into fatty acid methyl esters (FAMEs) with the basic method using boron trifluoride according to the Polish standards (PN-EN ISO 12966-2: 2011). The FAME composition was analyzed by gas chromatography with mass spectrometer (Clarus 600 GC/MS system, PerkinElmer, USA) equipped with an Elite-5MS capillary column (length: 60 m; inner diameter: 0.25 mm; film thickness: 0.25 µm). Helium with a constant flow of 1 mL min${}^{-1}$ was used as the carrier gas. The sample volume was 1 mL (split ratio, 50:1). The injector temperature was 290 ${}^{\circ }$C. The column started at a temperature of 110 ${}^{\circ }$C and was ramped up to 180 ${}^{\circ }$C at a rate of 5 ${}^{\circ }$C per minute, then 15 min at 180 ${}^{\circ }$C, followed by the gradient of 5 ${}^{\circ }$C per minute up to 290 ${}^{\circ }$C and then 5 min at this temperature. The temperature of transfer line was 290 ${}^{\circ }$C. For mass spectrometry, the selected-ion recording technique was used with the ionization energy of 70 eV and an ion source temperature of 200 ${}^{\circ }$C. The individual FAMEs were identified by the comparison of their retention times with that of the standard compound (Supelco^™^ 37 Component FAME Mix, Sigma-Aldrich, Germany). A total of 37 fatty acids were investigated in milk samples. However, only fatty acids with an even number of carbon atoms were considered in the association analyses, since only these are synthesized de novo, elongated, and desaturated in the mammary gland. The peaks were analyzed with TurboMass software (PerkinElmer Inc., Waltham, MA, USA).

### 2.4 Statistical analysis

Statistical analyses were performed using the appropriate R packages (R Core Team, 2015). An additive relationship matrix was constructed based on a three-generation pedigree using the kinship2 R package (Therneau et al., 2014). The following linear model (Eq. 1) was constructed and estimated using the lmekin function of the coxme R package (Therneau, 2015):

1 $$Y=\mu \;+\;G\;+\;\mathrm{LS}\;+\;\beta_{1}A\;+\;\beta_{2}\mathrm{DIM}\;+\;\alpha \;+\;e,$$

where Y is the phenotypic value of each trait, μ is the overall mean, G is the fixed effect corresponding to the genotype of polymorphisms, LS is the fixed effect of lactation number and lactation season, $\beta_{1}$ is the regression coefficient for cow age (A), $\beta_{2}$ is the regression coefficient for days in milk (DIM), α is the random polygenic effect for all known pedigree relationships, and e is the random residual.

## 3 Results

### 3.1 SNP identification and genotyping

**Figure 1:**
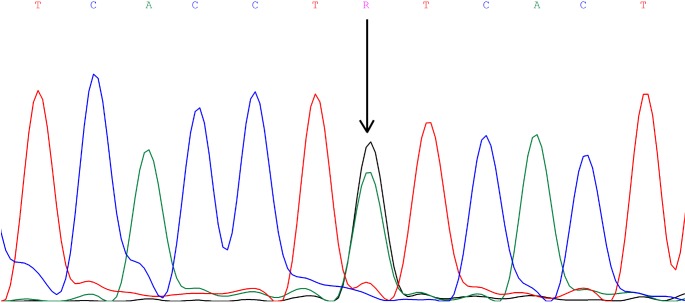
The rs209202414 A-to-G polymorphism in intron 3 of the bovine *FADS2* gene revealed by DNA sequencing.

**Figure 2:**
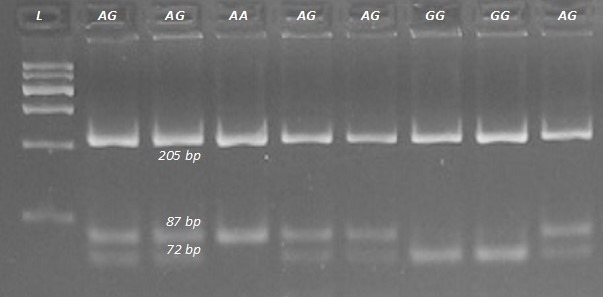
Genotyping the rs209202414 A-to-G polymorphism in the bovine *FADS2* gene. Digestion with *TseF*I restriction enzyme revealed the *AA*, *AG*, and *GG* genotypes. L is a 600 bp DNA ladder.

DNA fragments overlapping exons 1, 3, and 12 with the parts of adjacent introns of the *FADS2* gene were sequenced. These analyses revealed the presence of an A to G substitution at position 23 of intron 3 (Fig. 1; GenBank rs209202414). The PCR products amplified with the F3 and R3 primers (Table 1) were digested with *Tse*FI restriction enzyme. After electrophoresis, the following genotypes were observed: *GG* (205, 72, 15 bp), *AG* (205, 87, 72, 15 bp), and *AA* (205, 87 bp). The 15 bp fragments were not detectable (Fig. 2). The following genotype distributions were observed: for Jerseys, *AA* = 0.24, *AG* = 0.63, and *GG* = 0.13; for Polish Holstein-Friesians, *AA* = 0.17, *AG* = 0.40, and *GG* = 0.43. According to the chi-squared test, these distributions differed significantly ($\chi^{2}=25.63$; P<0.01). In the Jersey group, the major allele was A, while in the Polish Holstein-Friesian group, the G allele was prevalent (Table 2).

**Table 2:** Genotypic frequencies of the rs209202414 SNP in the bovine *FADS2* gene.

		Genotype			Allele	
Breed	n	*AA*	*AG*	*GG*	A	G
Jersey	104	0.24	0.63	0.13	0.55	0.45
Holstein-Friesian	150	0.17	0.40	0.43	0.37	0.63

### 3.2 The association of genotype with fatty acid composition in the milk of Polish Holstein-Friesian and Jersey cattle

Fatty acid composition in the milk fat of Jersey and Polish Holstein-Friesian cows is given in Tables 3 and 4. The association analysis indicated significant differences in some FA content between cows carrying different *FADS2* genotypes. In Jersey cattle, significant associations were recorded between the *FADS2* (rs209202414) polymorphism and the following milk FA: lauric (P=0.0486), behenic (P=0.0199), lignoceric (P=0.0209), oleic (P=0.0386), eicosatrienoic (P=0.0113), and docosadienoic (P=0.0181). In Polish Holstein-Friesian cows, significant associations were observed for erucic (P=0.0460) and docosahexaenoic (P=0.0469) acids.

**Table 3:** The association of *FADS2* polymorphism with the fatty acid composition (%) in the milk of Jersey cows.

Trait		Total (n = 104)	Genotype			P
			*AA*	*AG*	*GG*	
			(n = 25)	(n = 65)	(n = 14)	
MY	Milk yield (kg)	21.381 ± 3.945	20.048 ± 3.354	21.698 ± 3.763	22.286 ± 5.295	n.s.
FY	Fat yield (kg)	1.065 ± 0.213	1.028 ± 0.186	1.071 ± 0.215	1.108 ± 0.251	n.s.
FP	Fat (%)	5.018 ± 0.706	5.182 ± 0.791	4.953 ± 0.676	5.024 ± 0.689	n.s.
C6:0	Caproic	2.705 ± 0.388	2.666 ± 0.326	2.699 ± 0.405	2.8 ± 0.423	n.s.
C8:0	Caprylic	1.692 ± 0.285	1.645 ± 0.2	1.695 ± 0.306	1.763 ± 0.317	n.s.
C10:0	Capric	3.467 ± 0.447	3.397 ± 0.412	3.487 ± 0.479	3.495 ± 0.363	n.s.
C12:0	Lauric	4.001 ± 0.55	3.914${}^{a}$ ± 0.514	4.026${}^{b}$ ± 0.588	4.044 ± 0.43	0.0486
C14:0	Myristic	12.459 ± 1.172	12.356 ± 1.132	12.514 ± 1.24	12.384 ± 0.947	n.s.
C16:0	Palmitic	37.721 ± 2.864	37.369 ± 2.926	37.979 ± 2.955	37.148 ± 2.297	n.s.
C18:0	Stearic	12.6 ± 1.645	12.556 ± 1.498	12.625 ± 1.813	12.562 ± 1.043	n.s.
C20:0	Arachidic	0.107 ± 0.02	0.108 ± 0.013	0.107 ± 0.021	0.109 ± 0.026	n.s.
C22:0	Behenic	0.028 ± 0.007	0.027${}^{a}$ ± 0.006	0.028 ± 0.007	0.03${}^{b}$ ± 0.009	0.0199
C24:0	Lignoceric	0.021 ± 0.006	0.02${}^{a}$ ± 0.004	0.021 ± 0.006	0.023${}^{b}$ ± 0.007	0.0209
C14:1	Myristoleic	1.333 ± 0.367	1.422 ± 0.399	1.305 ± 0.346	1.306 ± 0.404	n.s.
C16:1	Palmitoleic	1.589 ± 0.374	1.634 ± 0.386	1.565 ± 0.345	1.623 ± 0.488	n.s.
C18:1n-9c	Oleic	16.487 ± 3.085	17.105${}^{a}$ ± 3.39	16.152${}^{b}$ ± 3.125	16.938 ± 2.147	0.0386
C18:1n-9t	Elaidic	1.036 ± 0.189	0.993 ± 0.185	1.049 ± 0.199	1.053 ± 0.143	n.s.
C18:2n-6c	Linoleic	1.997 ± 0.342	1.992 ± 0.326	1.995 ± 0.357	2.013 ± 0.321	n.s.
C18:3n-3	α-Linolenic	0.115 ± 0.052	0.112 ± 0.046	0.118 ± 0.055	0.106 ± 0.045	n.s.
C18:3n-6	γ-Linolenic	0.013 ± 0.004	0.012 ± 0.004	0.013 ± 0.005	0.013 ± 0.004	n.s.
C20:1	Eicosenoic	0.006 ± 0.002	0.007 ± 0.002	0.006 ± 0.002	0.006 ± 0.002	n.s.
C20:2	Eicosadienoic	0.006 ± 0.002	0.007 ± 0.001	0.006 ± 0.002	0.006 ± 0.002	n.s.
C20:3n-3	Eicosatrienoic	0.08 ± 0.03	0.076 ± 0.025${}^{a}$	0.082 ± 0.032${}^{b}$	0.082 ± 0.03	0.0113
C20:3n-6	Eicosatrienoic	0.01 ± 0.004	0.01 ± 0.003	0.01 ± 0.004	0.011 ± 0.005	n.s.
C20:4n-6	Arachidonic	0.125 ± 0.036	0.125 ± 0.031	0.124 ± 0.037	0.131 ± 0.043	n.s.
C20:5n-3	Eicosapentaenoic	0.023 ± 0.007	0.022 ± 0.005	0.024 ± 0.007	0.025 ± 0.008	n.s.
C22:1n-9	Erucic	0.025 ± 0.007	0.024 ± 0.005	0.025 ± 0.007	0.027 ± 0.008	n.s.
C22:2	Docosadienoic	0.002 ± 0.001	0.002${}^{a}$ ± 0.001	0.002 ± 0.001	0.003${}^{b}$ ± 0.001	0.0181
C22:6n-3	Docosahexaenoic	0.002 ± 0.001	0.002 ± 0.001	0.002 ± 0.001	0.002 ± 0.001	n.s.
C24:1	Nervonic	0.004 ± 0.001	0.004 ± 0.001	0.004 ± 0.001	0.004 ± 0.001	n.s.

${}^{a,\;b}$ Different superscripts within rows indicate statistically significant differences (p≤0.05). n.s. – non-significant.

**Table 4:** The association of *FADS2* polymorphism with the fatty acid composition (%) in the milk of Polish Holstein-Friesian cows.

Trait		Total (n = 150)	Genotype			P
			*AA*	*AG*	*GG*	
			(n = 25)	(n = 60)	(n = 65)	
MY	Milk yield (kg)	30.842 ± 8.402	31.54 ± 7.276	30.78 ± 8.004	30.631 ± 9.229	n.s.
FY	Fat yield (kg)	1.267 ± 0.357	1.25 ± 0.259	1.288 ± 0.362	1.254 ± 0.387	n.s.
FP	Fat (%)	4.147 ± 0.627	4.036 ± 0.695	4.207 ± 0.55	4.134 ± 0.668	n.s.
C6:0	Caproic	2.25 ± 0.437	2.314 ± 0.377	2.199 ± 0.416	2.272 ± 0.476	n.s.
C8:0	Caprylic	1.309 ± 0.245	1.349 ± 0.244	1.307 ± 0.242	1.296 ± 0.251	n.s.
C10:0	Capric	3.053 ± 0.56	3.076 ± 0.488	3.091 ± 0.595	3.01 ± 0.556	n.s.
C12:0	Lauric	3.694 ± 0.643	3.717 ± 0.597	3.731 ± 0.706	3.652 ± 0.605	n.s.
C14:0	Myristic	12.33 ± 1.444	12.418 ± 1.246	12.229 ± 1.571	12.391 ± 1.407	n.s.
C16:0	Palmitic	41.182 ± 4.881	41.62 ± 4.92	41.386 ± 5.032	40.826 ± 4.775	n.s.
C18:0	Stearic	9.209 ± 2.518	8.714 ± 1.878	9.414 ± 2.83	9.21 ± 2.432	n.s.
C20:0	Arachidic	0.054 ± 0.019	0.05 ± 0.011	0.055 ± 0.02	0.054 ± 0.02	n.s.
C22:0	Behenic	0.017 ± 0.007	0.016 ± 0.005	0.016 ± 0.007	0.019 ± 0.009	n.s.
C24:0	Lignoceric	0.016 ± 0.01	0.014 ± 0.005	0.014 ± 0.006	0.018 ± 0.013	n.s.
C14:1	Myristoleic	1.361 ± 0.49	1.394 ± 0.45	1.325 ± 0.476	1.381 ± 0.522	n.s.
C16:1	Palmitoleic	2.175 ± 0.702	2.028 ± 0.531	2.17 ± 0.783	2.236 ± 0.68	n.s.
C18:1n-9c	Oleic	16.523 ± 3.259	16.558 ± 3.435	16.258 ± 3.472	16.753 ± 3.013	n.s.
C18:1n-9t	Elaidic	0.994 ± 0.297	1.015 ± 0.319	0.993 ± 0.263	0.987 ± 0.322	n.s.
C18:2n-6c	Linoleic	2.822 ± 0.659	2.754 ± 0.603	2.823 ± 0.564	2.848 ± 0.761	n.s.
C18:3n-3	α-Linolenic	0.282 ± 0.094	0.262 ± 0.079	0.285 ± 0.09	0.288 ± 0.102	n.s.
C18:3n-6	γ-Linolenic	0.012 ± 0.004	0.012 ± 0.004	0.012 ± 0.004	0.012 ± 0.003	n.s.
C20:1	Eicosenoic	0.004 ± 0.002	0.004 ± 0.002	0.004 ± 0.002	0.004 ± 0.002	n.s.
C20:2	Eicosadienoic	0.004 ± 0.002	0.004 ± 0.002	0.004 ± 0.002	0.004 ± 0.002	n.s.
C20:3n-3	Eicosatrienoic	0.066 ± 0.026	0.063 ± 0.02	0.068 ± 0.029	0.065 ± 0.026	n.s.
C20:3n-6	Eicosatrienoic	0.007 ± 0.003	0.007 ± 0.003	0.007 ± 0.003	0.008 ± 0.003	n.s.
C20:4n-6	Arachidonic	0.139 ± 0.043	0.14 ± 0.027	0.141 ± 0.05	0.137 ± 0.043	n.s.
C20:5n-3	Eicosapentaenoic	0.02 ± 0.006	0.02 ± 0.005	0.02 ± 0.006	0.02 ± 0.006	n.s.
C22:1n-9	Erucic	0.022 ± 0.016	0.02 ± 0.015	0.019${}^{a}$ ± 0.014	0.025${}^{b}$ ± 0.018	0.046
C22:2	Docosadienoic	0.004 ± 0.003	0.003 ± 0.002	0.003 ± 0.002	0.004 ± 0.003	n.s.
C22:6n-3	Docosahexaenoic	0.001 ± 0.002	0.001 ± 0.001	0.003${}^{a}$ ± 0.001	0.002${}^{b}$ ± 0.003	0.0469
C24:1	Nervonic	0.004 ± 0.004	0.003 ± 0.001	0.004 ± 0.001	0.005 ± 0.005	n.s.

${}^{a,b}$ Different superscripts within rows indicate statistically significant differences (p≤0.05). n.s. – non-significant.

## 4 Discussion

The most important non-genetic factor significantly affecting fat content, and milk fatty acid profile in particular, is nutrition. It is associated with the fact that the fats contained in the feed are in part the source of fatty acids in the milk of ruminants, which are also the product of the reactions occurring in the rumen and lactocytes (Palmquist, 2006). The fatty acid composition in the milk samples of cows in the present study corresponded with the results obtained for dairy breeds. The most represented group of FAs in milk was SFA, followed by monounsaturated fatty acid (MUFA) and PUFA, which is consistent with the previous results (Carvajal et al., 2016; Hanuš et al., 2016; Vranković et al., 2017). Vranković et al. (2017) showed a similar FA composition in the milk of Holstein cows (C10:0 = 3.053, C12:0 = 3.694, C14:0 = 12.33 vs. C10:0 = 3.00, C12:0 = 3.70, C14:0 = 12.03) at the 150th day of lactation.

Ibeagha-Awemu et al. (2014) found significant associations of several polymorphisms in the *FADS* cluster with oleic acid, AA, dihomo-γ-linolenic acid (DGLA), SFA, and MUFA indices, but not with C20:5n-3, C20:5n-6, or C22:6n-3 in the milk of Canadian Holstein cows. The authors suggested a possible involvement of these SNPs in FA synthesis and indicated them as potential genetic markers in the breeding programs increasing the content of milk FAs that are valuable for human health.

Oleic acid is a health-beneficial product of the delta-9 desaturation of stearic acid, catalyzed by SCD. Burdge and Wootton (2002) demonstrated that docosahexaenoic acid (DHA) is produced internally through a series of desaturation and elongation reactions from the dietary precursor, α-linolenic acid. The positive effect of DHA on health has been extensively reviewed (Calder and Yaqoob, 2009; Ponnampalam et al., 2018). However, no associations were found between the *FADS2* polymorphism and the C18:2n-6c, C18:3n-3, and D6D indices. This may be a result of dietary PUFA precursor (LA and ALA) susceptibility to biohydrogenation in the rumen (Chikunya et al., 2004). Appropriate supplementation of dairy cow diets may change the proportion between milk SFA and MUFA/PUFA concentrations. In the study by Kliem et al. (2019) on the use of whey protein and rapeseed oil gel as feed supplements in Holsteins, an incremental inclusion of whey protein gel caused a linear increase in MUFA and PUFA and the same decrease in SFA. Bougouin et al. (2019), investigating an effect of starch-rich or lipid-supplemented diets in lactating Holstein cows, found a higher milk SFA concentration and lower MUFA and trans-10 C18:1 concentrations in the animals fed diets containing the Ca salts of palm oil and starch from maize grain and wheat in comparison with those comprising extruded rapeseeds and sunflower seeds, whereas the levels of trans-11 C18:1 were unchanged. Finally, Santillo et al. (2016) observed an increased level of SFA, MUFA (mainly due to the contribution of C18:1 cis-9), and PUFA in Italian Simmental cows supplemented with dietary whole flaxseed.

The PUFA level in an organism is related to many positive health outcomes and plays a crucial role in its function. Some of these effects are determined by the LC-PUFA (Tosi et al., 2014). Animals are unable to synthesize essential fatty acids (EFAs), but they can convert them (from the diet) to more unsaturated FA with a longer carbon chain (Nakamura and Nara, 2004). The desaturation and elongation processes of omega-3 acids are carried out by desaturases and elongases leading to the formation of LC-PUFA (Cormier et al., 2014).

In humans, Al-Hilal et al. (2013) reported that the *FADS* polymorphisms are very important regulators of LC-PUFA synthesis and explained the variance of several fatty acids. Similar results were published by Boschetti et al. (2015), who demonstrated relationships between genotype and desaturating ability and, consequently, a significant impact on the PUFA content in poultry meat. Fast-growing chickens showed lower expression of hepatic *FADS1* and *FADS2* and thus a significantly lower content of, for example, 18:2(n-6) and 20:4(n-6) FA (P<0.01) in breast meat. Other factors can also modulate FADS2 activity. Cho et al. (1999) showed that dietary PUFA can abolish the level of hepatic *FADS2* mRNA in human. Takeuchi et al. (2010) reported that a high level of dietary PUFA can suppress the transcription of *SREBP-1c*, a major transcription factor involved in the upregulation of *FADS2* expression. Diet components may affect *SREBP1* expression or activity. In the study by Li et al. (2018) on fatty acid composition in the muscles of Yanbian Yellow steers, the expression of *SREBP1* increased with age in the animals fed a corn-based finishing diet with an increasing proportion of corn in the ration (every 4 months). Han et al. (2012), investigating the expression of lipogenic genes in lactating Holsteins, found that the expression of *SREBP1* in the mammary gland was downregulated in the animals fed the Ca salts of conjugated linoleic acid (CLA), whereas Harvatine and Bauman (2006) reported that treatments causing milk fat depression (in the form of a low forage, high oil diet, and the trans-10, cis-12 CLA infusions) decreased the expression of *SREBP1* in the bovine mammary gland.

In beef cattle, Matsumoto et al. (2014) found a significant effect of the SNP (g.-823G > A) in the promoter region of the *FADS2* gene on carcass traits and fatty acid composition. In Japanese Black cattle, the percentage of C14:0 in the *GG* animals was higher than that of the *GA* ones. Subcutaneous fat thickness of the *GG* individuals was thinner than that of the *GA* ones, which led to higher yield estimates for the former. The beef marbling score of Holstein animals carrying the *GG* genotype was significantly higher than that of the *GA* individuals. An analogous relationship (although non-significant) was observed in Japanese Black cattle. Finally, the percentage of C16:0 was higher for the *GG* genotype compared with the *GA* genotype, and the percentage of MUFA was higher in the *GA* animals than that in the *GG* animals with a higher percentage of SFA in the latter. A later study on Japanese Black steers (Takahashi et al., 2016) showed that a highly significant association existed between the rs211580559 SNP (C > T in exon 7) and intramuscular C18:2(n-6) composition (with the *CC* individuals having significantly higher C18:2(n-6) composition than the *CT* ones), whereas no significant relationships between this SNP and other investigated fatty acids (C14:0, C14:1, C16:0, C16:1, C18:0 and C18:1) were found. Beak et al. (2019) analyzed an SNP (rs109772589) in the *FADS2* gene for its possible association with the fatty acid profile in Hanwoo beef cattle. However, all genotyped animals had the same *GA* genotype. Therefore, no further analysis was performed.

The differences in the milk FA profile between Jersey and Polish Holstein-Friesian cows may be determined by inter-breed variations in milk FA composition, which has been previously reported (Palladino et al., 2010). A nutrigenomic study showed that cows fed ALA- or LA-rich diets had increased PUFA and decreased SFA levels in milk compared with a control diet, which resulted from a diet-specific differential regulation of genes involved in FA metabolism in the mammary gland. The authors postulated that a lower level of SFA was due to the suppression of genes involved in FA metabolism and synthesis, and a higher level of PUFA was a consequence of the increased availability and incorporation of substrates used for milk PUFA synthesis (Ibeagha-Awemu et al., 2016). Different genetic variants may affect the level of FA or indices. In the study by Ding et al. (2016) on the role of selected SNP in the *FADS* gene cluster (*FADS1*, *FADS2*, and *FADS3*) on the PUFA concentration in the breast milk of Chinese women, the rs1535 SNP and two-locus haplotypes in the *FADS2* gene as well as a two-locus haplotype in the *FADS1* gene were associated with the GLA and AA concentrations with the minor allele carriers having lower concentrations of these acids. On the other hand, the three-locus haplotype in the *FADS2* gene significantly affected concentrations of GLA but not AA. The cited authors also showed that the individuals homozygous for an SNP in the *FADS3* gene had lower concentrations of ALA and LA in their breast milk. Mychaleckyj et al. (2018), investigating breast milk fatty acid composition in Bangladeshi mothers, showed that AA is the primary FA in breast milk influenced by genetic variation at the *FADS1*/*2*/*3* locus and that the most significant genetic association at this locus was with the fraction of AA at the rs174556 SNP. Finally, Kgwatalala et al. (2009) reported that one of the analyzed regulatory variants in the *SCD1* gene was associated with higher C10 and C12 desaturase indices and higher contents of C10:1 and C12:1 in the milk of Holstein cows.

In recent years, there have been several genome-wide association studies on milk fat traits (Grisart et al., 2002; Daetwyler et al., 2008; Moioli et al., 2007). The majority of associated SNPs were located in intergenic and intronic regions (Ibeagha-Awemu et al., 2016). Intronic SNPs may affect highly conserved elements and cis-acting RNA, which can impact RNA splicing and the rate of mRNA transcription (Millar et al., 2010; Hong et al., 2018).

## 5 Conclusions

This study showed a significant association between the *FADS2* polymorphism and milk fatty acid composition in Jersey and Polish Holstein-Friesian cattle. The differences between breeds may result from the inter-individual variation in milk FA metabolism. The study indicated the A-to-G substitution (rs209202414) in the bovine *FADS2* gene as a potential genetic marker for fatty acid composition in cattle milk.

## Data Availability

The data used in the present study are confidential and therefore not publicly available.
